# Inhibition of Chloride Intracellular Channel 1 (CLIC1) as Biguanide Class-Effect to Impair Human Glioblastoma Stem Cell Viability

**DOI:** 10.3389/fphar.2018.00899

**Published:** 2018-08-21

**Authors:** Federica Barbieri, Roberto Würth, Alessandra Pattarozzi, Ivan Verduci, Chiara Mazzola, Maria G. Cattaneo, Michele Tonelli, Agnese Solari, Adriana Bajetto, Antonio Daga, Lucia M. Vicentini, Michele Mazzanti, Tullio Florio

**Affiliations:** ^1^Sezione di Farmacologia, Dipartimento di Medicina Interna and Centro di Eccellenza per la Ricerca Biomedica, Università di Genova, Genova, Italy; ^2^Dipartimento di Bioscienze, Università degli Studi di Milano, Milan, Italy; ^3^Dipartimento di Biotecnologie Mediche e Medicina Traslazionale, Università degli Studi di Milano, Milan, Italy; ^4^Dipartimento di Farmacia, Università di Genova, Genova, Italy; ^5^IRCCS, Ospedale Policlinico San Martino, Genova, Italy

**Keywords:** glioblastoma, cancer stem cells, metformin, biguanides, proliferation, CLIC1

## Abstract

The antidiabetic biguanide metformin exerts antiproliferative effects in different solid tumors. However, during preclinical studies, metformin concentrations required to induce cell growth arrest were invariably within the mM range, thus difficult to translate in a clinical setting. Consequently, the search for more potent metformin derivatives is a current goal for new drug development. Although several cell-specific intracellular mechanisms contribute to the anti-tumor activity of metformin, the inhibition of the chloride intracellular channel 1 activity (CLIC1) at G1/S transition is a key events in metformin antiproliferative effect in glioblastoma stem cells (GSCs). Here we tested several known biguanide-related drugs for the ability to affect glioblastoma (but not normal) stem cell viability, and in particular: phenformin, a withdrawn antidiabetic drug; moroxydine, a former antiviral agent; and proguanil, an antimalarial compound, all of them possessing a linear biguanide structure as metformin; moreover, we evaluated cycloguanil, the active form of proguanil, characterized by a cyclized biguanide moiety. All these drugs caused a significant impairment of GSC proliferation, invasiveness, and self-renewal reaching IC_50_ values significantly lower than metformin, (range 0.054–0.53 mM vs. 9.4 mM of metformin). All biguanides inhibited CLIC1-mediated ion current, showing the same potency observed in the antiproliferative effects, with the exception of proguanil which was ineffective. These effects were specific for GSCs, since no (or little) cytotoxicity was observed in normal umbilical cord mesenchymal stem cells, whose viability was not affected by metformin and moroxydine, while cycloguanil and phenformin induced toxicity only at much higher concentrations than required to reduce GSC proliferation or invasiveness. Conversely, proguanil was highly cytotoxic also for normal mesenchymal stem cells. In conclusion, the inhibition of CLIC1 activity represents a biguanide class-effect to impair GSC viability, invasiveness, and self-renewal, although dissimilarities among different drugs were observed as far as potency, efficacy and selectivity as CLIC1 inhibitors. Being CLIC1 constitutively active in GSCs, this feature is relevant to grant the molecules with high specificity toward GSCs while sparing normal cells. These results could represent the basis for the development of novel biguanide-structured molecules, characterized by high antitumor efficacy and safe toxicological profile.

## Introduction

Novel drug development is currently facing significant drawbacks due to the increasing costs of preclinical screening and the more stringent rules on drug safety adopted by government agencies. Thus, a system already intrinsically inefficient (i.e., few molecules every year can be proved to perform better than existing drugs) is now becoming economically unsustainable (Paul et al., 2010; Avorn, 2015; Dovrolis et al., 2017). This is particularly true for cancer treatment, where costs afforded for the development of targeted therapies impede their availability for all the potentially responsive patients. In this scenario, drug repositioning (Langedijk et al., 2015; Sleire et al., 2017) represents one of the new strategies used to maximize the efficiency of the drug development process, allowing a faster and less expensive bench-to-clinic translation, and to minimize risks of unexpected toxicities (Kim et al., 2016; Nosengo, 2016; Wurth et al., 2016). This approach is aimed to identify novel therapeutic indications for drugs previously approved for different uses. In particular, drugs, already gone through clinical trials, either currently in clinical use for different diseases or discontinued for inefficacy on their original indication, can be repurposed for novel therapeutic approaches.

Glioblastoma (GBM, WHO grade IV astrocytoma) is a rapidly growing brain tumor characterized by invasive and pro-angiogenic behavior leading to poor prognosis (Brat et al., 2007; Furnari et al., 2007; Louis et al., 2007). GBM is still an incurable tumor and, notwithstanding the development of new treatments, including a multimodal approach in which maximal safe surgery is followed by radiotherapy and temozolomide as cytotoxic drug, the median survival rate is still about only 15 months (Stupp et al., 2009). In fact, due to the infiltrating nature, a complete surgical resection of GBM is virtually impossible and the tumor rapidly relapses. Other possible reasons responsible for the therapeutic failure of GBM treatments involve anatomical issues (i.e., the presence of the blood–brain barrier), the high neuronal toxicity of treatments, and, more importantly, the presence of small populations of cancer cells, named cancer stem cells (CSCs), which are highly refractory to radiotherapy and cytotoxic drugs (Vescovi et al., 2006; Lathia et al., 2011; Meyer et al., 2015). CSCs play a primary role in the development and recurrence of most of solid and hematological tumors including GBM. Typically, but not always, CSCs are characterized by CD133 expression as surface marker (Ludwig and Kornblum, 2017), and, similarly to normal stem cells, persist within the tumor mass due to their self-renew ability. CSCs are able to differentiate giving origin to all the phenotypes composing the bulk of the different cell populations within the tumor. Thus, the presence of CSCs confers great cell heterogeneity to GBM and, importantly, resistance to chemo- and radio-therapy involving different molecular mechanisms (Singh et al., 2004; Florio and Barbieri, 2012; Park et al., 2018). In fact, like normal stem cells, CSCs display high expression of DNA repairing enzymes and drug efflux transporters. Recently, several reports support the notion that GBM CSCs (GSCs) derive not only from transformed neural stem or progenitor cells but also from the de-differentiation of non-CSC “differentiated” tumor cells in response to micro-environmental factors (Friedmann-Morvinski, 2014; Suva et al., 2014). Therefore, CSCs are nowadays considered the crucial players for the development of several neoplastic diseases, including GBM, and represent an elective target to obtain efficacious therapeutic responses. However, to date, only few reports have identified, at a preclinical level, a significant antitumor activity on human GSCs of known or experimental drugs (Griffero et al., 2009; Gupta et al., 2009; Sachlos et al., 2012; Carra et al., 2013; Triscott et al., 2015; Angeletti et al., 2016), making this tumor cell population a relevant target to test repositioned drugs. In recent years, several molecules were repositioned as anticancer agents [i.e., disulfiram, itraconazole, aspirin, sertraline, ritonavir, propranolol, among others (Wurth et al., 2016)], but metformin is the best studied and most promising drug candidate (Wurth et al., 2014; Chae et al., 2016; Heckman-Stoddard et al., 2017; Romero et al., 2017).

Metformin is a biguanide derivative used as first-line drug for treating type 2 diabetes. Currently, millions of people are treated with metformin showing only minor adverse reactions. Starting from epidemiologic studies, a potential chemopreventive effect of metformin was identified in diabetic patients, demonstrating a significant lower incidence of several solid tumors (Evans et al., 2005). Following this evidence, numerous preclinical studies demonstrated that metformin significantly reduce cell proliferation in different *in vitro* and *in vivo* cancer models, including breast (Hirsch et al., 2009; Barbieri et al., 2015; Baldassari et al., 2018), prostate (Ben Sahra et al., 2011; Kato et al., 2015), colon (Zaafar et al., 2014), neuroblastoma (Costa et al., 2014), osteosarcoma (Gatti et al., 2016, 2018; Xu et al., 2017; Paiva-Oliveira et al., 2018), and, relevant to this study, GBM (Sato et al., 2012; Wurth et al., 2013; Yang et al., 2016; Kim et al., 2017). Notably, while displaying toxic effects in several tumor cells, metformin is basically harmless for normal stem cells (Wurth et al., 2013; Gritti et al., 2014), confirming the safety profile of this drug as observed after chronic use in diabetic patients. Similar antitumor effects have also been reported for other structurally-related biguanides, in particular phenformin and buformin (Zhu et al., 2015; Jiang et al., 2016; Petrachi et al., 2017; Rajeshkumar et al., 2017), two antidiabetic agents withdrawn from clinical use due to the risk of lactic acidosis. Moreover, experimental biguanides, never tested in clinics, were reported to exert *in vitro* antitumor activity in GBM and ovarian cancer cells (Choi et al., 2016; Zhang et al., 2016).

Mechanistically, several different intracellular signals were identified as potential mediators of metformin antitumor activity. First, it was proposed that metformin causes the activation of the AMP-activated protein kinase (AMPK), similarly to what observed in liver to inhibit glucose release (Rena et al., 2017). In turn, AMPK inhibits mTOR pathway causing cell growth arrest (Foretz et al., 2014), although recent studies proposed that, in GBM, the activation of AMPK could lead to increased proliferation (Chhipa et al., 2018). However, several other intracellular pathways endowed with a potential antiproliferative activity are affected by metformin in tumor cells (i.e., Akt, STAT3, miRNA deregulation, among others) (Bao et al., 2012; Wurth et al., 2013; Feng et al., 2014). Moreover, metformin indirect antitumor effects, such as the inhibition of the release or the activity of hormones, cytokines, or growth factors, have also been observed (Foretz et al., 2014; Vella et al., 2016; Zhu et al., 2016). Thus, different, and apparently unrelated mechanisms of action, have been identified in different tumor cells as responsible of metformin antiproliferative activity. However, the observation that, at least *in vitro*, structurally-related biguanides are endowed with similar antitumor activity raises the possibility that a “class-effect” could be at the basis of such an activity. If this is the case, all the different effects on intracellular transduction systems triggered by metformin in different tumor cells might be dependent on a master molecular mechanism shared by all biguanides, granting these molecules a selective functional toxicity toward cancer cells while sparing normal cells. Following this idea, we recently identified a novel transduction mechanism specifically responsible for the proliferation of human GSCs and involved in metformin antitumor activity: the inhibition of the chloride intracellular channel 1 (CLIC1) activity (Gritti et al., 2014). CLIC1 is a metamorphic protein mainly localized in the cytosol as inactive monomer that under stress conditions, and in particular during the G1/S phase transition of the cell cycle, translocates to the membrane where it induces chloride ion influx to trigger proliferation (Peretti et al., 2015). Importantly, differently from normal cells where only few channels are active, CLIC1 activity is increased in tumor cells, suggesting that CLIC1 could represent a tumor-specific drug target (Setti et al., 2013). We reported that metformin binds CLIC1 in the extracellular portion of the putative pore region, thus blocking chloride current and causing cell proliferation arrest (Gritti et al., 2014). As expected from the tumor selective CLIC1 activity, metformin effects on chloride influx and cell division occurred only in GSCs, but not in human umbilical cord mesenchymal stem cells (ucMSC) (Gritti et al., 2014).

On these premises, numerous clinical trials were started to test the antitumor activity of metformin in different human tumors, and several are still ongoing (Heckman-Stoddard et al., 2017). However, important issues and concerns about this off-label activity are still unsolved. In particular, extremely high drug concentrations (within the mM range) are required to induce antitumor effects in *in vitro* studies, although *in vivo* metformin intratumoral concentrations were reported to be several fold higher than in plasma (Nguyen et al., 2017; Baldassari et al., 2018). It was therefore proposed that protracted *in vivo* treatment using clinically reachable doses, can possibly induce antitumor effects (Gritti et al., 2014). Notwithstanding, novel derivatives, retaining the same efficacy and safety profile of metformin, but endowed with higher potency, are currently intensively searched.

In this study, we compared efficacy and potency as far as antitumor activity in human GSCs of known biguanides approved for different diseases. In particular, we tested phenformin, a withdrawn antidiabetic drug, moroxydine, a former antiviral agent, and the antimalarial agent proguanil, all showing a biguanide linear structure as in metformin, and cycloguanil, the active form of proguanil, which contains a cyclized biguanide moiety. Moreover, we tested the activity of phenformin, moroxydine, proguanil, and cycloguanil on CLIC1 activity to establish whether its inhibition may represent a biguanides’ pharmacological class effect. This is of particular relevance to pave the way to the development of therapeutically improved novel compounds acting through the same mechanism of action but showing better pharmacokinetic and pharmacodynamic characteristics in comparison to known biguanides.

## Materials and Methods

### Reagents and Antibodies

Metformin (1,1-dimethylbiguanide hydrochloride), phenformin [1-(2-phenylethyl)-biguanide hydrochloride], dissolved in water, and IAA94 (indanyloxyacetic acid 94), in ethanol, were from Sigma-Aldrich (Milan, Italy). Moroxydine, cycloguanil and proguanil, were synthesized according to the procedures previously reported (Curd and Rose, 1946; Modest, 1956; Shapiro et al., 1959) and stock solutions were prepared in DMSO. The correspondent amount of DMSO (for moroxydine, cycloguanil and proguanil, max 0.3%) or ethanol (for IAA94, max 0.2%) was added in the control points, although we observed that, at these concentrations, the vehicles did not modify any of the parameter tested. Growth factor-reduced Matrigel^TM^ was from BD Biosciences (Franklin Lakes, NJ, United States), type 1 rat tail collagen from SERVA Electrophoresis GmbH (Heidelberg, Germany), and methylcellulose from Sigma-Aldrich.

The following antibodies were used: anti-CLIC1 cl. F9 and cl. 356.1 (Santa Cruz Biotechnology, Dallas, TX, United States) for immunofluorescence and Western blot experiments, respectively; anti-GFAP, anti-nestin, anti-Oct4, anti-Sox2, and anti-MAP-2 (Abcam, Cambridge, United Kingdom); anti-Olig2 and anti-Sox2, (Merck Vimodrone, Italy); anti-mouse and anti-rabbit Alexa Fluor 488 and 568 secondary antibodies (Thermo Fisher Scientific, Carlsbad, CA, United States).

### Human GSC and ucMSC Cultures

#### Human GSCs

Cultures were obtained from post-surgical samples of GBM grade IV (WHO classification) derived from 7 patients (3 females and 4 males, age range: 40–73, average 62 years old) and coded as GBM1 to GBM7. Post-surgical samples were used after patients’ informed and written consent and Institutional Ethical Committee (IEC) approval. All patients underwent surgery at Neurosurgery Department (Ospedale Policlinico San Martino, Genova, Italy) and had not received therapy prior to the intervention. Detailed information on individual patients is reported in **Table 1**.

**Table 1:** Characteristics of patients and tumors from which glioblastoma stem cells were isolated.

Code	Age at surgery	Sex	WHO grade	OS (months)	Cerebra hemisphere/ lobe Localization	Meningeal infiltration	IHC	MIB-index (%)	Molecular Type	MGMT promoter methylation	Allelic Loss^∗^		Allelic Gain^∗^
											
											BAL	MAL	MAG
GBM 1	57	M	IV (primary)	9,5	Right/T-P Cortical	no	GFAP+	40	Neural	Met	CDKN2A, TP53	RET, PTEN, BRCA2, RBI, MIRN15A, DLEU1	EGFR, KIT, MET, SMO, BRAF
GBM 2	48	M	IV (primary)	14,4	Left/T Subcortical	no	GFAP+	60	Neural	UNMet	CDKN2A	RET, PTEN	EGFR, MET, SMO, BRAF, STK11, FKBP8, MYC, MDM2
GBM 3	40	F	IV (secondary, progression from oligodendro-glioma)	14,8	Right/F-T Cortical-subcortical	yes	GFAP+	30	Mesenchymal	ND	CDKN2A, PTEN		MDM4, AURKB, ERB2, TOP2a, STK11
GBM 4	71	F	IV (primary, multicentric)	7,3	Left/T-P-0 Cortical-subcortical	no	GFAP+	30	Neural	Met		BRAF, SMAD4	MDM2,TSC2
GBM 5	70	M	IV (primary)	37,9	Left/F Subcortical	no	GFAP+	40	ND	Met	CDKN2A	SMAD4	MET, SMO, BRAF, PTCH1, ABL1, TSC1, STK11, FKBPS AURKA
GBM g	73	F	IV (primary)	9,9	Left/T-P Cortical	no	GFAP+	10	ND	ND	ND	ND	ND
GBM 7	54	M	IV (primary)	U.K.	Left/T-P Cortical	no	GFAP+ (low)	40	ND	ND	ND	ND	ND

*M, male; F, female; OS, overall survival; GFAP, glial fibrillary acidic protein; T, temporal; P, parietal; F, frontal; O, occipital. BAL, biallelic DNA loss; MAL, monoallelic DNA loss; MAG, monoallelic DNA gain. Met, methylated; UNMet, Unmethylated. U.K., unknown; ND, not determined. ^∗^Performed on isolated GSCs.*

**FIGURE 1:**
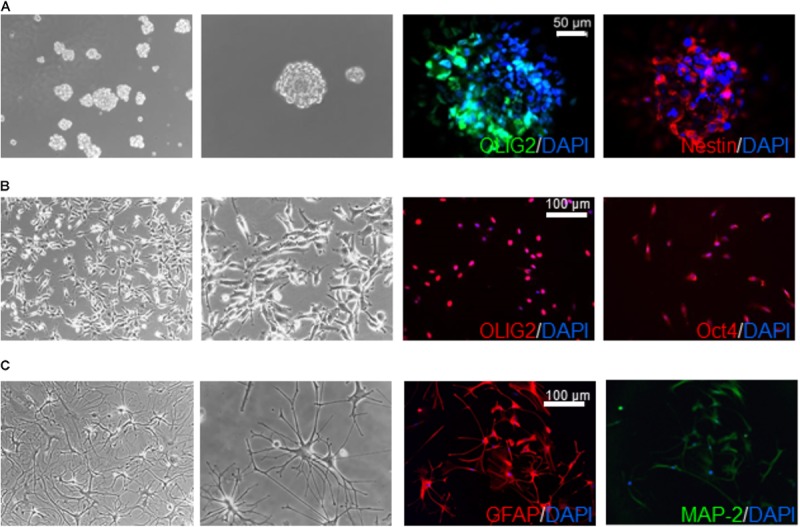
Characterization of human glioblastoma stem cells. Representative images of GSC (derived from GBM6) spheroids **(A)** and monolayer on Matrigel **(B)** cultured in stem cell medium supplemented with EGF and bFGF and differentiated cells **(C)** obtained by switching culture conditions from growth factor-deprived to FBS-containing medium. Left panels reproduce phase contrast images at different magnification (10× and 20×). Right panels depict immunofluorescence for stem cell markers OLIG2, nestin, and Oct4 **(A,B)**, and the glia (GFAP) and neuron (MAP-2) differentiation markers **(C)**. GFAP and MAP-2 expression is depicted from the same microscope field, showing in most cells the co-expression of both markers. Similar results were obtained analyzing all the GSC cultures that enter the study.

Glioblastoma stem cells, isolated as described (Gatti et al., 2013), were grown in stem cell-permissive medium enriched with 10 ng/ml human bFGF and 20 ng/ml human EGF (Miltenyi Biotec, Bergisch Gladbach, Germany) (Bajetto et al., 2013). Sphere formation occurred within 2 weeks of culture (**Figure 1**). For survival and electrophysiology experiments, GSCs were grown as monolayer on growth factor-reduced Matrigel coating, allowing easier evaluation without affecting stem cell features (Griffero et al., 2009). Validation of GSC properties of the cultures (i.e., self-renewal capacity, stem cell marker expression, multipotency, and tumorigenicity) was performed as described (Wurth et al., 2013) (**Figure 1**). All the cultures analyzed in this study have been characterized in previous works for tumor-initiating capacity by orthotopic injection of 10,000 sphere-derived cells in 6–8 weeks old non-obese diabetic severe combined immunodeficient (NOD/SCID) mice (Charles River Laboratories, Wilmington, MA, United States) (Carra et al., 2013; Wurth et al., 2013; Gritti et al., 2014; Corsaro et al., 2016). Cell differentiation was carried out by shifting GSC cultures in serum-containing medium (10% FBS) for at least 15 days (Griffero et al., 2009; Gatti et al., 2013).

Genetic alteration analysis in GSCs was performed by multiplex ligation-dependent probe amplification (MLPA) analysis. Briefly, genomic DNA was isolated from GSCs and normal human astrocytes (ScienCell Research Laboratories, Carlsbad, CA, United States) using QIAamp DNA microkit (Qiagen). The MLPA analysis (SALSA MLPA KITs P175-A1 Tumor-Gain and P294-A1 Tumor-loss, MRC Holland, Amsterdam, Netherlands) was performed using 100ng of genomic DNA, following the manufacturer’s instructions. The resulting DNA fragments were identified and quantified by using capillary electrophoresis on an ABI XL3130 genetic analyzer (Applied Biosystems, Foster City, CA, United States) and the Genemapper program (version 4.0 – Applied Biosystems). Data were analyzed with the Coffalyser software (MRC-Holland). For each GBM, gains and losses were assigned by comparing the peaks between the patient and the reference samples (DNA from normal human astrocytes), as previously described (Monticone et al., 2012, 2014).

CLIC1 gene down-regulation: short hairpins mRNA specific for human CLIC1 (5′-GATGATGAGGAGATCGAGCTC-3′) and firefly luciferase (5′-CGTACGCGGAATACTTCGA-3′) were cloned into the XhoI/HpaI sites of the pLentiLox 3.7 lentiviral vector and stably expressed in GBM2 CSCs, as reported (Setti et al., 2013; Gritti et al., 2014). CLIC1 downregulation was evaluated by Western blot (to monitor protein content) and by electrophysiology (to monitor channel activity).

#### Human ucMSCs

Cells were isolated from umbilical cords, obtained after cesarean section at Obstetrics and Gynecology Department of International Evangelical Hospital (Genova, Italy) following informed consent and approval by IEC, as reported (Bajetto et al., 2017). Briefly, after vessel removal, cords were treated with collagenase (0.5 μg/ml) to expose Wharton jelly and obtain single cells. Cells, grown in MesenPRO RS Medium^TM^ (Gibco, Thermo Fisher Scientific), underwent flow cytometry phenotypical characterization (MSC Phenotyping Kit, Miltenyi Biotec), showing that more than 95% of the cells, in each independent culture, were negative for hematopoietic antigens (CD45, CD34, CD14) and MHC class-II, but expressed CD73, CD105, CD90, CD29, and MHC class-I. Multipotency was demonstrated showing that after incubation in selective media, ucMSCs differentiate into osteocytic, chondrocytic, and adipocytic lineages (Bajetto et al., 2017).

### Immunofluorescence

Glioblastoma stem cells and differentiated GBM cells were fixed with 4% paraformaldehyde, permeabilized with PBS/0.1% Triton X-100, blocked with normal goat serum and immunostained with the appropriate antibody, followed by fluorochrome-conjugated secondary antibody (Wurth et al., 2017). Nuclei were counterstained with DAPI (Sigma-Aldrich). Slides were photographed with a DM2500 microscope (Leica, Milan, Italy) equipped with a DFC350FX digital camera (Leica) or confocal microscope (Bio-Rad MRC 1024 ES) (Corsaro et al., 2016).

### Western Blot Analysis

Cells were lysed in RIPA buffer and processed as reported (Florio et al., 2001). Proteins (20 μg), transferred to PVDF membrane (Bio-Rad Laboratories) and probed with primary antibodies, were then incubated with appropriated secondary antibodies and immunocomplexes detected by Clarity and Western ECL Substrates (Bio-Rad Laboratories).

### Cell Proliferation and Survival Assays

#### MTT Assay

Mitochondrial function, as index of cell viability, was evaluated by measuring the reduction of 3-(4,5-dimethylthiazol-2-yl)-2,5-diphenyltetrazolium bromide (MTT, Sigma-Aldrich), as reported (Porcile et al., 2014). Briefly, 4000 GSCs, differentiated GBM cells, or ucMSCs were treated with vehicle or the tested drugs for 48 h in dose-response experiments; afterwards, cells were incubated with 0.25 mg/ml MTT in serum-free medium for 2 h at 37°C; after the removal of the medium, formazan crystals were dissolved in DMSO and absorbance was spectrophotometrically measured at 570 nm, using an ELx800 microplate reader (BioTek, Winooski, VT, United States) (Villa et al., 2016a).

#### Cell Counting

Glioblastoma stem cells from different tumors, grown in standard conditions for 3 days in the presence or absence of vehicle or biguanides, were counted with an automated cell counter (TC20, Bio-Rad Laboratories, Inc., Hercules, CA, United States) every 24 h. Briefly, after treatments, cells were harvested and the suspension was diluted 1:10 in sterile PBS and mixed with an equal volume of 0.4% Trypan Blue solution. Cell viability was calculated as % of viable cells divided by the total number of cells (Villa et al., 2016b).

### Apoptosis Detection

Cells were treated with vehicle or the different biguanides at concentration corresponding to the respective IC_50_, and stained with Annexin V-FITC and propidium iodide (PI) (Apoptosis Detection kit; Thermo Fisher Scientific). Samples were analyzed by FACScalibur (BD Biosciences), equipped with BD CellQuest Pro software. The percentage of cell death was obtained by summing-up the percentages of early and late apoptosis (Pattarozzi et al., 2017).

### Evaluation of GSC Invasiveness

#### Chemoinvasion Assay

Experiments were performed in a 48-well modified Boyden chamber using 8 μm nuclepore polyvinylpyrrolidine-free polycarbonate filters, coated with Matrigel (100 μl/filter). Boyden chamber was assembled with the coated side of the filter facing the cells in the upper compartment. GSCs were suspended at a density of 1 × 10^6^ cells/ml in DMEM/F12 containing 0.1% bovine serum albumin, and 50 μl/well of the cell suspension were added to the upper chamber after 10 min pre-treatment with drugs or the corresponding vehicles. Drugs were present throughout the experiments. DMEM/F12 medium supplemented with 10% FBS was used as attractant in the lower chamber. After 6 h of incubation at 37°C, non-migrated cells on the upper surface of the filter were removed by scraping. The cells that had migrated to the lower side were stained with Diff-Quick stain (WVR, Radnor, PA, United States), and 5–8 unit fields per filter were counted by a scorer, blind to the experimental conditions, with a Zeiss microscope.

#### Three-Dimensional (3-D) Invasion Assay

Multicellular tumor spheroids were generated in the presence of methylcellulose as previously described (Cattaneo et al., 2012), with minor modifications. Briefly, GSCs were suspended at a density of 25,000 cells/ml in DMEM/F12 containing 1% FBS and 3% methylcellulose, 200 μl/well of the cell suspension were seeded in non-adherent round bottom 96-well plates and centrifuged for 10 min at 400 ×*g* immediately after plating. Tumor spheroids were harvested within 24 h, suspended in DMEM/F12 containing 10% FBS and 0.9% methylcellulose, and mixed with an equal volume of a collagen stock solution prepared by mixing at 4°C acidic rat tail collagen (2 mg/ml; 8 vol) with 1 vol of 10× DMEM and 0.1 M NaOH, to adjust pH to 7.4. Drugs, or the corresponding vehicles, were added to suspended spheroids before embedding them into collagen. Spheroid-containing gel was rapidly transferred into pre-warmed 24-well plates, and incubated for 24 h at 37°C in 5% CO_2_. In-gel invasion was quantified by measuring the length of all of the processes originating from individual spheroids using the ImageJ software (United States National Institutes of Health, Bethesda, MA, United States). At least 10 randomly selected spheroids per experimental group were measured in each experiment.

### Sphere-Formation Assay

Glioblastoma stem cells were seeded in complete medium in the absence of Matrigel in 48-well plates at 1000 cells/well. After 24 h, cells were exposed to different concentration of biguanides according to the calculated IC_50_, and monitored for 5 days, to allow sphere generation. The number of spheres/well was quantified using a digital camera mounted on a transmitted light microscope to image each individual well, and visually calculated by three independent operators, as reported (Angeletti et al., 2016). To further demonstrate the inhibitory activity of biguanides on sphere-formation process, GBM-derived tumor-spheres, generated in the absence or presence of biguanides, were disaggregated into single cells and re-plated at 1000 cells/well in fresh medium. Spheres-formation capacity was re-evaluated after 5 days, (on day 10, secondary spheres) and further repeated to on day 15 (tertiary spheres).

### Electrophysiology

Patch electrodes (GB150F-8P with filament, Science Products) were pulled from hard borosilicate glass on a Brown-Flaming P-87 puller (Sutter Instruments, Novato, CA, United States) and fire-polished to a tip diameter of 1–1.5 μm and an electrical resistance of 5–7 MΩ. Patch-clamp electrophysiology was performed in perforated-patch whole cell configurations, as reported (Gritti et al., 2014).

The voltage protocol consisted of 800 ms pulses from −40 mV to +60 mV every 10 s. Current amplitude was measured as trace average between 700 and 750 ms. Patch clamp solutions were the following: bath solution (mM): 140 NaCl, 5 KCl, 10 HEPES, 1 MgCl_2_, 2 CaCl_2_, 5 D-Glucose, pH 7.4. Pipette solution (mM): 135 KCl, 10 HEPES, 10 NaCl, 1 MgCl_2_, 2 CaCl_2_, 5 D-Glucose, pH 7.4.

#### CLIC1 Current Detection

Chloride intracellular channel 1 activity was detected using electrophysiological measurements. Cells under continuous membrane current monitoring were constantly perfused with external solution at 1 ml/min. Once current recordings reached a constant value, cells were challenged with the different biguanides, followed by addition of IAA94 (100 μM) to get maximum inhibition (Tonini et al., 2000). Data reported in the box chart plots are the current ratio between the effect of each biguanide at the reported concentrations, and the residual IAA94-sensitive current.

### Statistical Analysis

All data are presented as means ± SEM. Statistical significance was established at *p*-value ≤ 0.05. Prism version 5.02 (GraphPad, San Diego, CA, United States) software was used to analyze the results. All experiments were repeated independently at least three times (*n*) each performed in triplicate or quadruplicate (as indicated). The half maximal inhibitory concentration IC_50_ was calculated using non-linear regression curve fit analysis selecting the log(drug) vs. response-variable slope (four parameters) equation Statistical significance between two independent groups was assessed by *t*-test (unpaired, two-tailed) or one-way ANOVA for multiple groups, followed by Tukey’s, Dunnett’s or Bonferroni’s *post hoc* tests (*post hoc* tests were only applied when ANOVA gave *p* < 0.05). Direct comparison between GSCs and differentiated GBM cells after biguanides treatment were performed using two-way ANOVA with interaction terms between cell type and concentration; this analysis was followed by *post hoc* Bonferroni test to determine at which concentration the effect of treatment was statistically significant.

## Results

### GSC Isolation, Culture, and Differentiation

Glioblastoma stem cells were isolated from seven human GBMs (see **Table 1** for patient and tumor characteristics). As expected, GBM analyzed were heterogeneous with 3 classified as neural (GBM1, 2, and 4) and 1 as “mesenchymal” (GBM 3) and only GBM2 showed unmethylated MGMT promoter. Similar to normal stem cells, isolated GSCs, cultured in chemically defined medium (without serum and containing EGF and bFGF), are able to grow *in vitro* as spheroids or loosely adherent clones expressing stem cell-related markers (i.e., OLIG2, nestin) (**Figure 1A**). Alternatively, to perform a better analysis of GSC pharmacological responses, cells were cultured on Matrigel in the same medium (Griffero et al., 2009; Gritti et al., 2014) thus allowing the proliferation as monolayer. Importantly, in both culture conditions, GSCs are able to self-renew, to retain stemness marker expression (i.e., OLIG2 and Oct4) (**Figure 1B**) and the tumorigenic ability [as we reported in previous studies (Carra et al., 2013; Wurth et al., 2013; Gritti et al., 2014; Corsaro et al., 2016)]. From a molecular point of view, loss of CDKN2a was detected in GSCs from GBM1, 2, 3, and 5, PTEN in GBM1, 2 and 3, TP53 only in GBM1; moreover, gain of EGFR was observed in GBM1 and 2, and of BRAF in GBM1, 2, and 5 (for more details see **Table 1**).

Glioblastoma stem cell cultures, shifted for 15 days in serum-containing medium, undergo astrocytic and/or neural differentiation (i.e., increased expression of GFAP and MAP-2, **Figure 1C**) representing an *in vitro* model of the bulk of heterogeneous non-stem cells in GBM mass.

### Biguanides Inhibit GSC Proliferation With Different Potency

**FIGURE 2:**
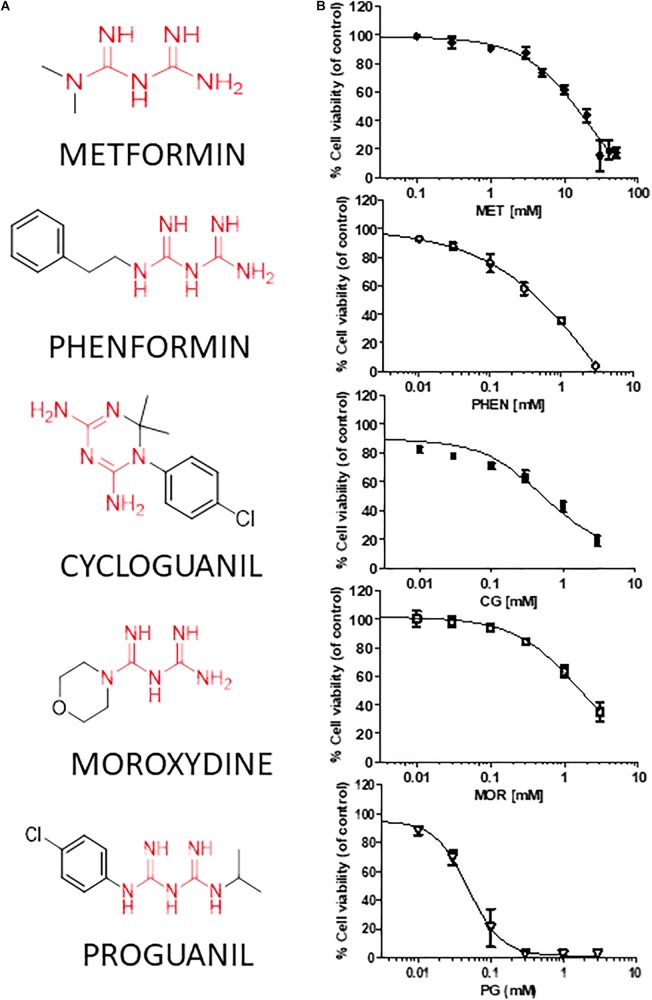
Metformin, phenformin, cycloguanil, moroxydine, and proguanil affect viability of glioblastoma stem cells. **(A)** Structural formulas of the compounds tested with the biguanide moiety highlighted in red. **(B)** Dose-response curves of the antiproliferative effect of biguanide derivatives on GBM CSC viability, evaluated by MTT assay after 48 h of treatment. Data were obtained from several GSC cultures, and represent the mean ± SEM of at least *n* = 3 independent experiments for each GBM, performed in quadruplicate on each culture. Metformin effects were tested in all the 7 GSC cultures, phenformin and cycloguanil in GBM1-6, moroxydine in GBM1-4 and 6, and proguanil in GBM2-4. Effects on individual GBM cultures are reported in **Supplementary Figure S1**. ^∗^*p* < 0.05, ^∗∗^*p* < 0.01, ^∗∗∗^*p* < 0.001 vs. CTR (ANOVA, Dunnett’s *post hoc* test).

We tested the effects of phenformin, cycloguanil, moroxydine, and proguanil on GSC viability in comparison with metformin. The molecular structures of the biguanides analyzed are reported in **Figure 2A**. In particular, we performed concentration-response experiments to determine both potency and efficacy of the different molecules. Growth inhibitory activity, measured by MTT assay after 48 h of treatment, was evaluated on GSCs grown on Matrigel. Indeed, we previously reported that metformin retains antiproliferative effects on GSCs, when grown either as spheroids or as monolayers (Wurth et al., 2013). The analysis was performed in seven individual GSC cultures (GBM1-7) to cope with GBM heterogeneity. Concentration-response curves, derived from the average results from all the culture tested, are reported in **Figure 2B** (curves of the effects of drugs on individual GBMs are reported in **Supplementary Figure S1**). In agreement with previous results, metformin caused a significant inhibition of GSC viability in all seven GBMs with a maximal inhibition of about 84% at 30 mM, and an average IC_50_ value of 9.4 mM (range: 2.1–12.9 mM) (**Figure 2B** and **Table 2**). Similar inhibitory effects were obtained with phenformin and cycloguanil on GBM1-6, and moroxydine on GBM1-4 and 6. However, phenformin, cycloguanil and moroxydine showed potencies more than 10-fold higher than metformin (0.35, 0.18, and 0.53 mM, respectively) (**Figure 2B** and **Table 2**). As far as efficacy, phenformin and cycloguanil showed a maximal inhibition of cell viability comparable to that of metformin (−96 and −81%, respectively, at 3 mM concentration), while moroxydine was less effective (−63%) (**Figure 2B** and **Table 2**). On the other hand, proguanil induced cytotoxicity with a low concentration-dependency, causing modest effects till 30 μM and an almost complete cell death at the concentration of 100 μM. Although the calculated IC_50_ was very low (54 μM), this curve is reminiscent of a non-specific cytotoxic effect (**Figure 2B** and **Table 2**).

**Table 2:** IC_50_ and efficacy values of the antiproliferative activity of different biguanides on GSC cultures isolated from individual human GBMs.

GSC code	Compound (mean IC_50_, mM)				
	*Metformin*	*Phenformin*	*Cycloguanil*	*Moroxydine*	*Proguanil*
**GBM1**	12.96	0.19	0.16	0.53	n.d.
**GBM2**	12.30	0.29	0.22	0.47	0.043
**GBM3**	6.22	0.60	0.15	0.54	0.034
**GBM4**	12.65	0.37	0.21	0.57	0.087
**GBM5**	2.10	0.20	0.21	n.d.	n.d.
**GBM6**	9.12	0.46	0.81	0.59	n.d.
**GBM7**	6.65	n.d.	n.d.	n.d	n.d.
**Average IC_50_** (mM ± SEM)	9.41 ± 1.54	0.35 ± 0.06	0.18 ± 0.10	0.53 ± 0.02	0.054 ± 0.016
**Efficacy**	84%	96%	81%	50%	98%
(% max cell death)[mM]	[30 mM]	[3 mM]	[3 mM]	[3 mM]	[1 mM]
**ucMSC** (mean IC_50_ mM ± SEM)	n.r	n.r.	n.r.	n.r.	0.048 ± 0.003

*IC_50_ values and the average efficacy (i.e., maximal inhibition of cell viability) of each compound is reported, as well as the effects on ucMSC viability. n.d., not determined; n.r., not reached.*

**FIGURE 3:**
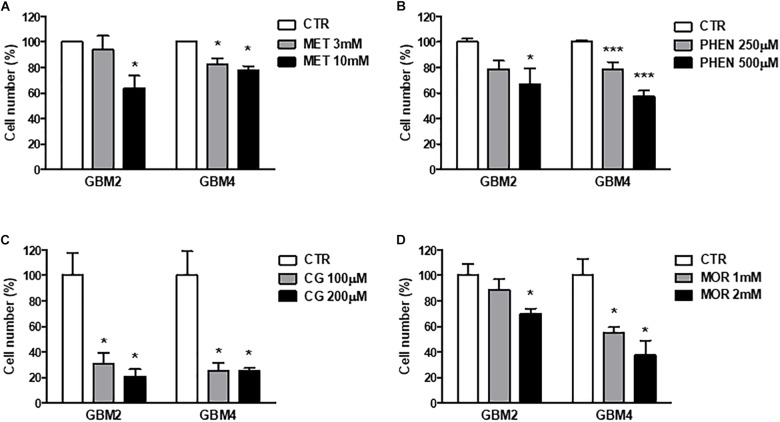
Metformin, phenformin, cycloguanil, and moroxydine exert antiproliferative activity on glioblastoma stem cells. Inhibition of GSC proliferation by metformin **(A)**, phenformin **(B)**, cycloguanil **(C)**, and moroxydine **(D)** was evaluated by counting number of cells after 48 h of treatment with various concentrations derived from the IC_50_ values, as calculated in **Table 2**. Experiments were performed on both GBM2 and GBM4 cultures, and data represent the average ± SEM of *n* = 3 independent experiments performed in quadruplicate. ^∗^*p* < 0.05, ^∗∗∗^*p* < 0.001 vs. respective CTR (ANOVA, Dunnett’s *post hoc* test).

Similar results were obtained measuring cell viability by Trypan blue exclusion test after treatment of GBM 2 and 4 CSCs for 48 h with the different molecules (**Figure 3**). The drugs were used at concentrations around the previously identified IC_50_ values. Also in these experiments cycloguanil confirmed to be the most potent and efficacious biguanide in contrasting GSC proliferation (**Figure 3C**).

**FIGURE 4:**
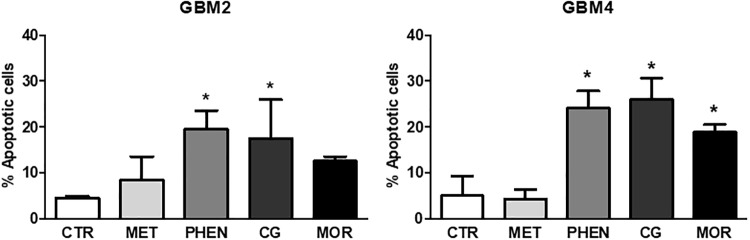
Pro-apoptotic effects of metformin, phenformin, cycloguanil, and moroxydine on glioblastoma stem cells. Induction of apoptosis by biguanide treatment for 48 h was detected by annexin V binding, measured by FACS in GBM2 **(left)** and GBM4 **(right)** CSCs. Metformin did not significantly increase apoptosis neither in GBM2 nor in GBM4. Phenformin and cycloguanil increased apoptotic cell number in both GBM2 and GBM4 CSCs, while moroxydine significantly affected only GBM4 CSCs. Bars represent the percentage of annexin V-positive cells ± SEM, counting 10,000 events *per* experimental points. Experiments were repeated twice using independent cell preparations. ^∗^*p* < 0.05, vs. CTR (*t*-test).

Next, we tested whether the antiproliferative effects of these biguanides could be ascribed to the activation of the apoptotic program by measuring annexin-V binding after 24 or 48 h of GBM2 and GBM4 CSC treatment. Metformin (10 mM) did not show pro-apoptotic activity after both 24 h (data not shown) and 48 h (**Figure 4**) of treatment. Similar results were also obtained using GBM3 cells (data not shown). A slight induction of apoptosis was observed in GBM2 and GBM4 CSCs after 24 h treatment with phenformin, cycloguanil, and moroxydine, although did not reach statistical significance (data not shown). Conversely, 48 h of treatment of GBM2 caused an increase of +15 (*p* < 0.05), +12.5 (*p* < 0.05), and +8% (n.s.) with phenformin, cycloguanil, and moroxydine, respectively (**Figure 4**), while a statistical significant increase in annexin-V positivity was measured in GBM4 CSCs treated with all the drugs (about +20% over control cells, reaching a significance of *p* < 0.05) (**Figure 4**). The specificity of the effects of the tested drugs against GSC subpopulation was further demonstrated showing that 24 h treatment of GBM1 and GBM2 cultures with metformin, phenformin, cycloguanil, and moroxydine significantly reduced the expression of Sox2 (**Supplementary Figure S2**), a specific GSC marker, which is down-regulated in differentiated GBM cells (Banelli et al., 2015).

### Biguanides Inhibit GSC Invasiveness

**FIGURE 5:**
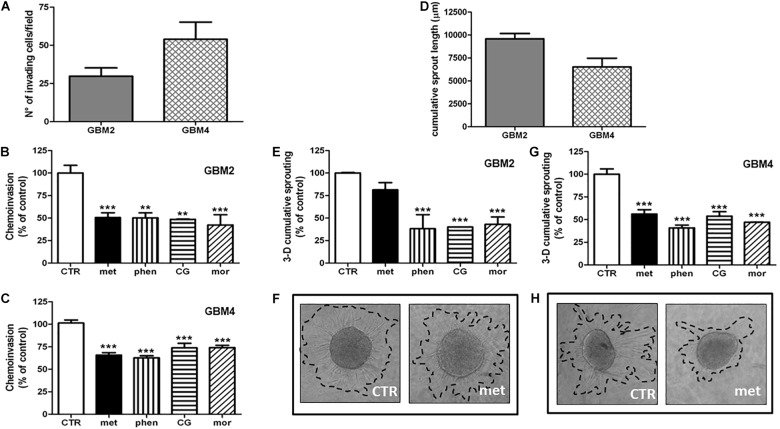
Biguanides inhibit glioblastoma stem cells invasiveness. **(A)** Basal GSC invasiveness was tested by measuring the number of invading GBM2 and GBM4 cells in chemo-invasion assays. Invading GBM2 **(B)** and GBM4 **(C)** cells were quantified in the absence (CTR) or in the presence of metformin (met, 10 mM), phenformin (phen, 500 μM), cycloguanil (CG, 100 μM) or moroxydine (mor, 1 mM). Data are expressed as the percent of the number of invading control cells (CTR) set at 100%. ^∗∗^*p* < 0.01, ^∗∗∗^*p* < 0.001 vs. CTR, *n* = 3. One-way ANOVA followed by Bonferroni’s *post hoc* test. **(D)** Cumulative length of invading sprouts emerging from spheroids obtained from GBM2 or GBM4 cells were quantified after 24 h of incubation as described in the “Materials and Methods” section. Cumulative length of invading sprouts emerging from GBM2 **(E)** and GBM4 **(G)** spheroids were quantified after 24 h of incubation in the absence (CTR) or in the presence of metformin (met, 10 mM), phenformin (phen, 500 μM), cycloguanil (CG, 100 μM), or moroxydine (mor, 1 mM). Data are expressed as the percent of the cumulative sprouting measured in control spheroids (CTR) set as 100%. ^∗∗∗^*p* < 0.001 vs. CTR, *n* = 3. One-way ANOVA followed by Bonferroni’s *post hoc* test. Representative images of GSCs sprouting outside the spheroids from control or metformin-treated GBM2 and GBM4 CSCs are shown in **(F,H)**, respectively, while representative images following the treatment with the other biguanides are reported in the **Supplementary Figure S2**.

Invasion of surrounding brain parenchyma is one of the main obstacle to the effective treatment of GBM. In chemoinvasion tests, we show that both GBM2 and GBM4 CSCs efficiently move through Matrigel (**Figure 5A**) and sprout out of spheroids in 3-D invasion assays (**Figure 5D**). We therefore tested the effect of metformin (10 mM), phenformin (0.5 mM), cycloguanil (0.1 mM), and moroxydine (1 mM) on the *in vitro* invasive properties of GBM2 and GBM4 cells. GSC invasion through Matrigel was significantly reduced by all the drugs (**Figures 5B,C**). No significant differences among metformin and the other biguanides were observed as far as the efficacy of the anti-invasive effects, ranging from about −50% to −30% of invading cells, in GBM2 and GBM4, respectively. However, the anti-invasive effects occurred at quite higher concentrations for metformin as compared to the other drugs, (**Figures 5B,C**), as already observed in cell viability assays.

Glioblastoma stem cell invasiveness was also tested in a 3-D assay on multicellular spheroids which more closely reproduce tissue-like morphology and cell-to-cell interactions. In these experimental conditions, phenformin, cycloguanil, and moroxydine significantly reduced the sprouting area of both GBM2 and GBM4 CSCs (about −60 and −50%, respectively) (**Figures 5E,G** and **Supplementary Figure S3** for representative images of GSCs sprouting out from spheroids). At variance, metformin efficiently reduced 3-D sprouting only in GBM4 (about −40%, **Figures 5G,H**) whereas it was much less effective in GBM2 cells, did not reaching statistical significance (−20% of control cells, **Figures 5E,F**).

### Biguanides Inhibit GSC Spherogenesis

**FIGURE 6:**
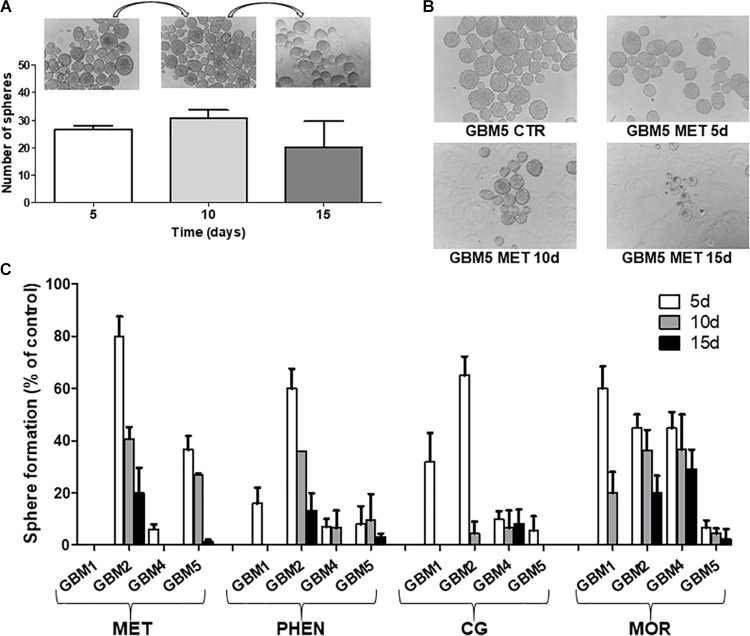
Biguanide treatment impairs sphere formation in glioblastoma stem cells. **(A)** GSC cultures possess self-renewal capability. Sphere formation assay was performed on four GSC cultures (GBM1, GBM2, GBM4, and GBM5); images of spheres were captured and counted as described in Section “Materials and Methods.” On day 5, primary spheres were dissociated into single cells and 1000 cells subjected to another round of sphere formation assay, counted on day 10 (secondary spheres) and re-plated for the third time to asses sphere formation on day 15 (tertiary spheres). Representative microphotographs (magnification 10×) and histograms quantifying sphere formation from GBM5 cells, are reported. Sphere formation assays were performed on GBM1, GBM2, GBM4, and GBM5 GSCs, and measurement of primary, secondary and tertiary sphere number was assessed on day 5, in control and cultures with metformin (MET, 10 mM), phenformin (PHEN, 0.25 mM), cycloguanil (CG, 0.2 mM), and moroxydine (MOR, 1 mM). **(B)** Representative microphotographs (magnification 10×) of GBM5 treated with metformin (10 mM) are reported. **(C)** Quantification of biguanide impairment of sphere formation in GBM1, GBM2, GBM4, and GBM5. Histogram depicts the time-course of the percentage of spheres developed in treated cultures after 5 days of exposure as compared to controls (5 days); after sphere disaggregation, single cells were grown in the absence of drugs, and the formation of secondary spheres was evaluated on day 10 and, following further sphere dissociation, on day 15.

Biguanide-induced changes of spherogenesis activity, a surrogate *in vitro* index of the self-renewal ability of GSCs (Soeda et al., 2008), were evaluated in four cultures (GBM1, 2, 4, and 5) to demonstrate the specific efficacy of the different drugs on GSCs. First we show that the GSC cultures used were able to self-renew. To do this, we allowed 1000 cells to form spheres for 5 days; these spheres were disaggregated and sequentially 1000 cells replated every 5 days. In untreated cells we observed an unaltered spherogenesis activity over the time (**Figure 6A**). The effects of metformin, phenformin, cycloguanil, and moroxydine (used at IC_50_-related concentrations) on self-renewal was tested treating 1000 cells at the moment of the first plating and evaluating after 5 days the number of spheres generated; the spheres were then disaggregated and 1000 cells replated in medium without drugs, and the formation of secondary spheres was measured in the following 5 days, this procedure was repeated for a third passage to test the ability of tertiary sphere formation capability of GSCs. In these experiments, although with different efficacy and with tumor-specific activity all the tested drugs were able to significantly reduce the number of cells able to generate new spheres (**Figures 6B,C**). In line with the previous experiments, moroxydine showed the lowest efficacy also on the inhibition of GSC self-renewal. Moreover, the different GBMs showed distinct sensitivity, with GBM1 being the more responsive (no spheres were generated after metformin treatment already after the first sphere formation assay on day 5, while phenformin and cycloguanil abolished spherogenesis after 10 days, and moroxydine after 15 days); conversely, GBM2 was the least sensitive since although reduced in number, few spheres were generated even at 15 days after metformin, phenformin and moroxydine treatment.

### Inhibition of CLIC1 Activity as Common Mechanism of the Antiproliferative Activity of Biguanides

**FIGURE 7:**
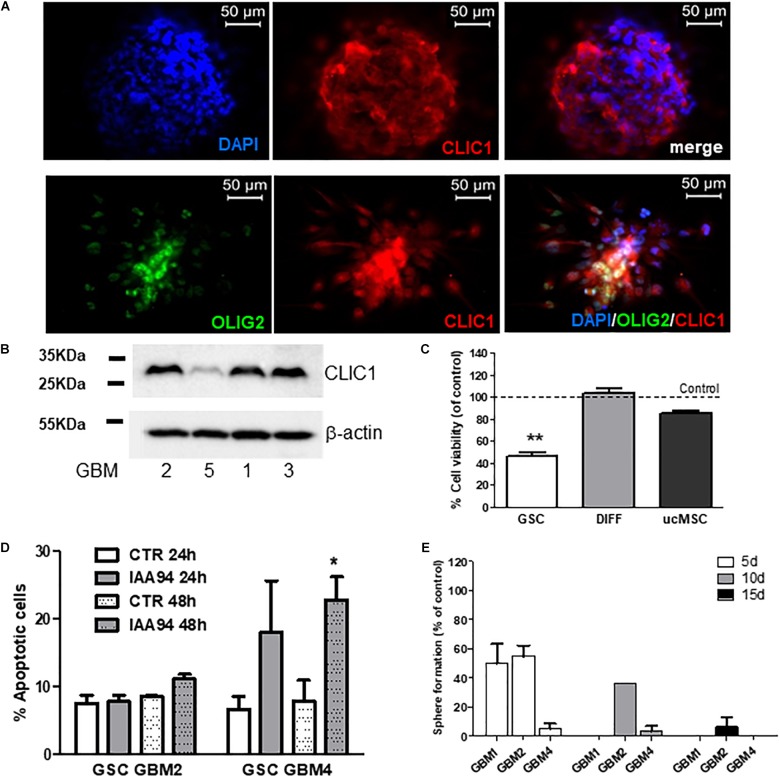
CLIC1 expression in glioblastoma spheroids and its role in cell viability, apoptosis, and self-renewal of glioblastoma stem cells. **(A)** CLIC1 expression was analyzed in GBM4 (upper) and GBM6 (lower) spheroids by immunofluorescence experiments. High expression of CLIC1 was detected in both spheroids, also showing a high level of co-localization with the stem cell marker OLIG2 (lower). **(B)** Representative Western blots showing the expression of CLIC1 in GSC cultures derived from GBM1, 2, 3, and 5. Beta-actin was used as reference housekeeping gene. **(C)** Effect of the CLIC1 inhibitor IAA94 (100 μM) on the viability of GSCs and their differentiated (DIFF) counterpart, and umbilical cord mesenchymal stem cells (ucMSC), measured by MTT assay. Only in GSCs, CLIC1 inhibition caused reduction of cell viability. Data are the mean of *n* = 2 experiments performed in quadruplicate, on each culture derived from 5 GSC cultures (GBM2, 3, 4, 5, 6), 3 differentiated cultures (GBM2, 3, and 4), and 3 independent ucMSC cultures. Dotted line represents control level set at 100%. ^∗∗^*p* < 0.01 vs. CTR cells. **(D)** Effect of IAA94 (100 μM) on the induction of apoptosis in GSCs from GBM2 and GBM4, measured by annexin V binding. A moderate pro-apoptotic effect (about 23% annexin V-positive cells) was observed only after 48 h of treatment in selected cultures (i.e., GBM4), while other cultures (GBM2) were not affected. Data are expressed as the mean of *n* = 3 independent experiments. ^∗^*p* < 0.05 vs. CTR (*t*-test). **(E)** Effect of IAA94 (100 μM) on GBM1, 2, and 4 spherogenesis. Cells were treated for 5 days with vehicle and IAA94 and the number of spheres counted and then disaggregated. 1000 cells were the replated and allowed to generate spheres for other 5 days (10 days) after then counted again, disaggregated and 1000 cells replated to form tertiary spheres for further 5 days (15 days).

We previously reported the role of CLIC1 membrane activity as selective and pivotal regulator of GSC proliferation. In agreement with these data, we confirmed that CLIC1 is enriched in GBM stem-like cells. In fact, CLIC1 was detected in spheroid cells derived from GBM2 and GBM4 which also co-express the neural stem cell marker OLIG2 (**Figure 7A**). Similar results, although showing different level of expression, were also obtained by Western blot analysis, in representative cultures, in **Figure 7B**. Moreover, the treatment of five GSC cultures with IAA94 (100 μM), a selective CLIC1 inhibitor (Valenzuela et al., 2000), reduced cell viability in all the cultures, as measured by MTT assay (**Figure 7C**). Conversely, IAA94 was ineffective in the correspondent differentiated cells from three of these GBM cultures and in three independent normal ucMSC cultures (**Figure 7C**), in line with the previous observations that in these cell types CLIC1 is dispensable for cell proliferation (Gritti et al., 2014). Finally, measuring annexin V binding by FACS, we observe that IAA94 (100 μM) treatment caused a low level of apoptosis in GBM4 (but not in GBM2) CSCs after 48h (**Figure 7D**), as we observed with the biguanide derivatives (see **Figure 4**). Finally, CLIC1 inhibition induced by IAA94 (100 μM) treatment significantly reduced the ability of GBM1, GBM2, and GBM4 to self-renew (**Figure 7E**), as assessed in a spherogenesis assay and detailed in the previous paragraph.

**FIGURE 8:**
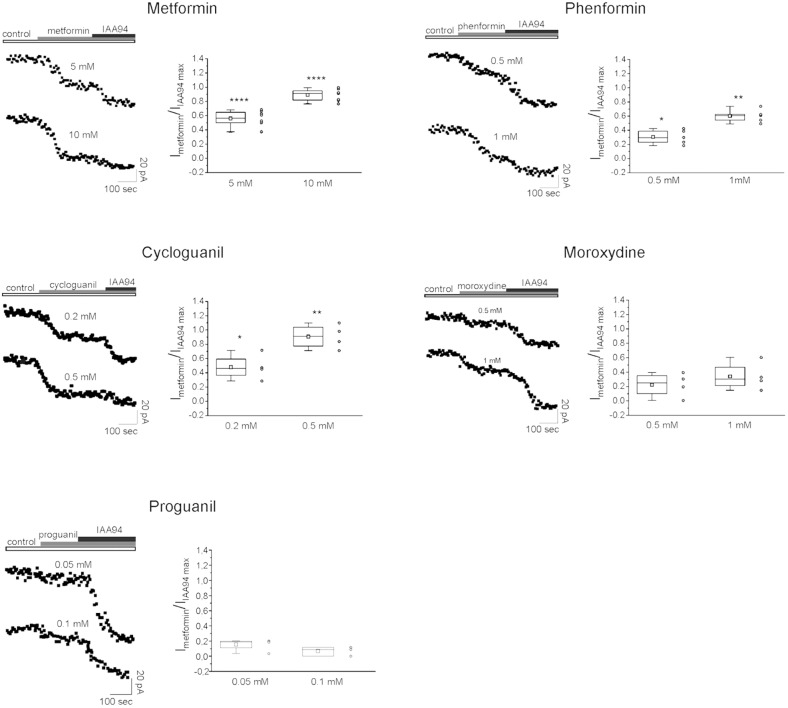
CLIC1 associated membrane current inhibition by biguanide compounds. Membrane current from GBM2 has been elicited by a 100 mV voltage step, from –40 to +60 mV, 800 ms in duration. Stimulus has been delivered every 10 s to generate a current amplitude time course. CLIC1 current inhibition is calculated as ratio of the effect induced by each compound and the inhibition observed after IAA94 (100 μM) treatment that represents the residual CLIC1 activity. For each compound are shown experimental data at two different concentrations reported on the top of each current time course **(left)**. The **(right)** depict box chart plots showing the average inhibition of CLIC1 current, calculated as ratio between the compound and IAA94 sensitive currents. Number of cells used for the statistics range from 4 to 8 for each compound concentration (empty circles next to box charts). Unpaired *t*-test: ^∗^*p* < 0.05, ^∗∗^*p* < 0.01, ^∗∗∗∗^*p* < 0.0001 vs. CTR cells.

Thus we checked whether the antiproliferative activity of the tested biguanides was dependent on a direct inhibition of CLIC1-associated membrane chloride current, as observed using metformin (Gritti et al., 2014). To test the activity and the relative potency of the different drugs, patch-clamp voltage-clamp experiments were performed in both GBM2 (**Figure 8**) and GBM4 CSCs (**Supplementary Figure S3**). Cells from each of these cultures were challenged in current time-course experiments in the presence of the biguanides at two different concentrations referred to the IC_50_ values determined in cell viability experiments. Data are reported as time-dependent current values during the sequential perfusion with vehicle (control), biguanides and IAA94. A correspondent box chart plots reports the percentage of inhibition induced by biguanides as ratio of the current block operated by IAA94, used at a concentration (100 μM) that completely inhibits CLIC1 activity (Tonini et al., 2000; Valenzuela et al., 2000) and represents CLIC1 current not affected by the tested drugs. Metformin caused a powerful inhibition of CLIC1 current (i.e., a very small reduction of residual membrane current was induced after adding IAA94 in metformin-treated cells). Similarly, also phenformin, cycloguanil and moroxydine inhibited CLIC1-associated ion current, in a concentration dependent manner. However, the potency differences observed in the viability assays (see **Table 2**) were confirmed in electrophysiology experiments: a maximal inhibition of CLIC1 activity was obtained with metformin at the concentration of 10 mM, with 1 mM phenformin, 0.5 mM cycloguanil, and 1 mM moroxydine (**Figure 8** and **Supplementary Figure S4**). Moreover, since phenformin did not cause a complete inhibition of the current (as shown by the additive inhibitory effect induced by IAA94 in the presence of the biguanides), we propose that this molecule might exert antiproliferative effects also via CLIC1-independent pathways. Moroxydine showed an incomplete CLIC1 inhibition but also a lower reduction of GSC viability, indicating a low efficacy as CLIC1 inhibitor and as antiproliferative agent for GSCs, but confirming the relationship between CLIC1 inhibition and antiproliferative effects. Because cycloguanil caused a complete inhibition of CLIC1 current at concentrations correspondent to the antiproliferative IC_50,_ it is evident that it is the most selective CLIC1 current inhibitor among all the tested compounds. The relevance of this mechanisms in the antiproliferative activity of the tested biguanides was further shown evaluating their antiproliferative activity in GBM2 CSCs (siCLIC1 cells) in which CLIC1 expression was down-regulated (−60% of both protein levels and channel activity, **Supplementary Figures 5A,B**). It was not possible to completely abolish CLIC1 gene expression since, in these conditions, cells are not viable. As compared to cells transfected with the empty vector (siLuc cells), in siCLIC1 cells the efficacy of all the drugs was significantly reduced (**Supplementary Figure 5C**). However, we do not have a complete abolishment of the antiproliferative activity of the drugs, especially with the drugs more effective in the inhibition of CLIC1 such as metformin, since the inhibition of the residual activity of the channel still causes a reduction of CSC proliferation.

Importantly, proguanil (50 and 100 μM, **Figure 8**) did not affect CLIC1 activity, clearly indicating that the high level of cytotoxicity observed in the previous experiments was independent from the blockade of CLIC1-mediated membrane chloride current, the molecular target of the other biguanides, and it is likely attributable to an *in vitro* non-specific effect.

**FIGURE 9:**
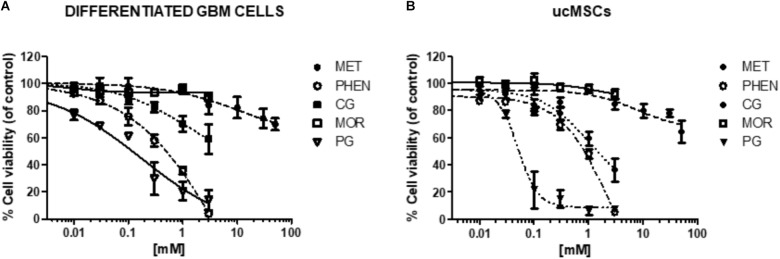
Effect of biguanides on the viability of cells in which CLIC1 associated membrane current is not relevant for cell proliferation. Concentration-response curves of metformin (MET), phenformin (PHEN), cycloguanil (CG), moroxydine (MOR), and proguanil (PG) in differentiated GBM cells **(A)** and ucMSCs **(B)**. Cell viability was assessed by MTT reduction test, after 48 h of treatment. Data are the mean of *n* = 3 experiments performed in quadruplicate, on cells derived from 5 GBMs (for metformin: GBM1, 2, 3, 4, 6, 7) and 3 GBMs (for phenformin, cycloguanil, moroxydine, and proguanil: GBM2, 3, 4) and 4 independent ucMSC cultures. For sake of clarity statistical significance is reported in **Supplementary Table S1**. The comparison of the effects of biguanides in GSCs and differentiated cells from the same GBM is reported in **Supplementary Figure S6**.

On the other hand, we confirmed that the effects of the biguanides on CLIC1 activity was merely functional since we did not observe changes in the channel expression after treatment with metformin, phenformin, cycloguanil, moroxydine, and IAA94, used as control compound (**Supplementary Figure S6**).

### Specificity of CLIC1 Inhibition in the Antiproliferative Activity of Biguanides

To test whether the antiproliferative activity of biguanides is specifically mediated by targeting CLIC1 activity, we measured the effects of the different biguanides on the viability of cells in which it was previously demonstrated that CLIC1 activity is not necessary for cell cycle progression (Wurth et al., 2013; Gritti et al., 2014). In particular, we tested the biguanides on the proliferation of differentiated GBM cells (obtained from the same GSC cultures previously analyzed by shifting cells in FBS-containing medium, see **Figure 1**), and ucMSCs. In both cell types we previously reported low levels of CLIC1-dependent ion current (Gritti et al., 2014). In fact, IAA94, used at 100 μM, a concentration that completely blocks CLIC1 activity (Tonini et al., 2000), did not affect viability of both differentiated GBM cells or ucMSCs (see **Figure 7B**). In agreement with previous data (Wurth et al., 2013; Gritti et al., 2014), metformin marginally affects differentiated GBM cell viability (−20% vs. controls) only when used at extremely high concentrations (i.e., 50 mM, **Figure 9A** and **Supplementary Table S1** for statistical significance). Conversely, phenformin caused a significant reduction of cell viability starting from concentrations around the IC_50_ value (0.3 mM), and the toxic effect further increased in a concentration-dependent manner reaching a maximal effect at 3 mM (**Figure 9A** and **Supplementary Table S1**). Cycloguanil did not show toxic effects on differentiated GBM cells for concentrations up to the IC_50_ identified for the antiproliferative activity (about 0.3 mM), but induced a moderate level of cell death at 1 and 3 mM (−30 and −48% viability vs. control cells). Conversely, moroxydine did not affect differentiated GBM cell at all concentrations tested. Finally, proguanil caused a dose-dependent cell death in differentiated GBM cells, starting at low concentrations (−31% cell viability at 0.03 mM) reaching more than 85% loss of viability at the highest concentration tested (3 mM, **Figure 9A** and **Supplementary Table S1**). Comparing the activity of metformin, phenformin, cycloguanil, and moroxydine in GSCs and differentiated cells from the same tumor, we show a statistically significant higher inhibition of the biguanides in GBM2, 3 and 4 (*p*-values ranged from <0.0001 to <0.001 for the effect of the drugs in the different cell types, see **Supplementary Figure S5**) with the exception of phenformin and moroxydine in GBM4, in which, however, although dose-response curves were not statistically different a significant difference was observed for the highest concentrations tested in the two cell populations (**Supplementary Figure S7**).

The same approach was used to investigate biguanide toxicity on ucMSCs (**Figure 9B** and **Supplementary Table S1**). Also in this case metformin and moroxydine did not affect cell viability at all the concentrations tested. Phenformin confirmed a dose-dependent toxicity that started to be statistically significant at the concentration of 0.3 mM, and cycloguanil was ineffective at the IC_50_-related concentrations but caused ucMSC death for concentrations about 10-fold higher than the IC_50_ value. The cytotoxic effect of proguanil on ucMSC was similar to that observed in GSC cultures showing an “all-or-nothing” effect, with a significant cytotoxicity already evident at the concentration of 0.1 mM (**Figure 9B** and **Supplementary Table S1**).

## Discussion

Repositioning of metformin as antitumor drug represents one of the hot topics in pharmacological oncology (Coyle et al., 2016). Epidemiological and preclinical studies provided solid bases to start clinical trials for different cancer histotypes, using metformin either as single or as chemo/radio-sensitizing agent. Unfortunately, to date, no clear results were attained in clinical studi as well as to search and develop improved metformin derivatives. Thus, metformin studies were redirected “from bed to bench.” In particular, two main issues have been brought to the attention of researchers: the high concentrations required to obtain antitumor effects *in vitro* and the lack of a defined mechanism by which metformin interferes with tumor cell proliferation. This is particularly relevant for GBM, since, among several other mechanism of action recently identified, metformin was more often reported to exert antiproliferative effects through the activation of AMPK and the subsequent inhibition of mTOR, a mechanism shown to promote GBM CSC growth (Chhipa et al., 2018; Leli and Koumenis, 2018). Thus a different intracellular mechanism should be regulated by metformin, and possibly other biguanides, to affect the proliferation and survival of this specific GBM cell subpopulation.

In this study, searching for more efficacious and potent metformin derivatives, we tested the effects of different biguanide-based molecules on GSC survival and/or proliferation, invasiveness and self-renewal; we also evaluated the selectivity of the biguanides toward tumor cells (and on the CSC component, in particular) in comparison to normal stem cells, to verify the safety profile as already reported for metformin. Specifically, we searched for a common intracellular mechanism, targeted by all the biguanides, underlying their antitumor effects, since the identification of a pharmacological class-effect might pave the way for novel, more potent and efficacious biguanide-based drugs to be tested in the clinical setting. Our results show that, beside metformin, other conformationally different biguanide derivatives exert antiproliferative and anti-invasive effects on GSCs, being also able to powerfully inhibit the self-renewal (as for the spherogenesis assay) ability of this GBM cell subpopulation, supporting their potential use as repositioned antitumor agents. Although all these molecules are known to act through the modulation of different intracellular pathways, we observed that the inhibition of GSC proliferation by all these drugs is mainly mediated by the blockade of CLIC1 ion conductance, which we propose as main mechanism of action responsible for the antitumor effects of all the molecules containing a biguanide moiety. Interestingly, a similar effect of metformin on CLIC1 activity was independently reported in gallbladder cancer cells (Liu et al., 2017), further confirming our evidence. At molecular level, we previously reported that the biguanide moiety of metformin is able to interact with a critical arginine (Arg29) in the CLIC1 putative pore region on the extracellular side, to prevent the activation of the ion current (Gritti et al., 2014). Therefore, our data support the hypothesis that CLIC1 inhibition represents a pharmacological class-effect of compounds featured by a biguanide substructure, which is the main determinant of cell proliferation arrest. Thus, both linear (metformin, phenformin, or moroxydine) or cyclized biguanides (cycloguanil) share the same mechanism of CLIC1 inhibition. Interestingly, we observed a rather significant relationship between the level of CLIC1 inhibition and the antiproliferative activity exerted by the drugs on GBM CSCs. For example, moroxydine, showing the lower efficacy as GSC growth inhibitor (see **Table 2**), caused only a partial blockade of the CLIC1-associated current. Conversely, a high inhibition of GSC proliferation, as observed using metformin and cycloguanil, correlates with a complete inhibition of CLIC1 activity, although the latter drug was much more potent than the former one (IC_50_: 9.4 vs. 0.18 mM). Importantly, we observed that all the compounds tested display a higher potency than metformin to inhibit GBM CSC proliferation, reaching IC_50_ about 100-fold lower. This observation suggests that these molecules could be effective *in vivo* at more easily reachable doses. Another difference among the compounds tested is the ability of some of these drugs to promote GSC apoptosis. Phenformin and cycloguanil caused a pro-apoptotic effect albeit modest (+15 and +13%, respectively); moroxydine effects were even lower, and not statistically significant in all the cultures. Moreover, a pro-apoptotic activity was never observed in metformin-treated cells. Since also CLIC1 inhibition induced by IAA94 mainly results in mainly a cytostatic effect with low level of induction of apoptosis in some cultures, we hypothesize that although metformin is the more CLIC1-selective drug among all the biguanides tested, also the other compounds largely mimic the activity of the CLIC1 inhibitor.

Glioblastoma heterogeneity is a relevant issue for the prognosis and the treatment options for this tumor. In fact, recently, different GBM subtypes, based on molecular features have been identified (Lieberman, 2017). Interestingly, although the analysis is rather incomplete due to the limited number of cases studied, we did not observe differences in the drug response according to genetic characteristics of the GBMs (i.e., MGMT promoter methylation, loss of PTEN, CDKN2a, gain of EGFR, etc.). Although larger studies are required, this observation suggest that the inhibition ofCLIC1 may represent a target common to most GBM subtypes to impair CSC proliferation.

We also compared the effects of biguanides on the viability of differentiated GBM cells and ucMSCs, which are not dependent on CLIC1 activity to proliferate. In fact, in these cell types CLIC1 inhibition by IAA94 does not reduce proliferation, and we previously reported that CLIC1 activity is almost undetectable, at least in the experimental conditions adopted in our study (Gritti et al., 2014). Thus, we assume that the effects observed in both differentiated GBM cells and ucMSCs can be classified as “non-specific” effects (i.e., CLIC1-independent toxic activity). In both these cell populations, a “non-specific” inhibition of viability was induced at all concentrations tested by proguanil, which indeed is unable to inhibit CLIC1 activity, by high concentrations of phenformin, and, at a lower extent, by maximal cycloguanil concentrations. The occurrence of “non-specific” cytotoxic effects might explain why phenformin, although unable to completely block CLIC1 currents at concentrations higher (i.e., 1 mM) than the antiproliferative IC_50_ (0.35 mM), still causes high level of GSC viability inhibition. Being the high specificity of metformin (and moroxydine) toward GSCs (due to the selective CLIC1 inhibition) a likely determinant of its harmless effects in normal cells (as demonstrated by its large use as frontline antidiabetic drug), we propose that a higher specificity for CLIC1 inhibition might grant selected biguanides with lower unwanted toxicities.

In agreement with this assumption, proguanil, which resulted highly toxic in all the tested cell types (GSCs, differentiated GBM cells, and ucMSC), showed a rather non-specific (i.e., CLIC1-independent) mechanism of action. This observation could appear in contrast with the clinical use of this molecule as antimalarial drug. However, it has to be pointed out that upon absorption proguanil is partially converted to cycloguanil by liver (Wangboonskul et al., 1993) and it is unlikely that the concentrations we used *in vitro* can be reached in most patients’ tissues. Moreover, off-target toxicities have been reported in proguanil-treated individuals (mouth ulcers, vasculitis, etc.). Notwithstanding, we analyzed proguanil *in vitro* effects mainly to identify biguanide structures able or not to exert a specific inhibition of CLIC1 activity. In particular, from this analysis we observed that proguanil is not able to inhibit CLIC1 activity, probably as a result of its chemical structure, which combines the simultaneous presence of 1-(4-chlorophenyl) ring and of the bulky 5-isopropyl group on the rigid biguanide skeleton (see **Figure 1A**), thus possibly preventing the access to CLIC1 pore in the extracellular region.

In conclusion, we identified the inhibition of CLIC1 activity as biguanide class-effect mediating antiproliferative effects in GSCs. Considering the selective role of CLIC1 in controlling cell cycle progression in GSCs (Verduci et al., 2017), we propose that the broad antitumor activity of metformin and related compounds, which has been reported in a high number of different human tumors, might depend on the interference with the chloride current associated to this protein. In fact, since increased CLIC1 activity is a main regulator of cell proliferation in several tumors, including gastric, colon, and hepato-carcinomas (Peretti et al., 2015), and, of course, GBM (Wang et al., 2012; Setti et al., 2013; Gritti et al., 2014), this mechanism may reconcile the evidence of antitumor activity of metformin (and possibly of other biguanides) on such broad types of neoplasia.

Importantly, we identify several related molecules which, although completely different from a structural point of view (both linear and cyclized biguanides), show higher potency than metformin, reaching as in the case of cycloguanil, about 50-fold lower IC_50_, and possibly are able to overcome the high concentrations required to induce antiproliferative effects, the major limitation in metformin clinical usefulness as anticancer drug. Furthermore, we also characterized the tested biguanides for efficacy and selectivity toward CLIC1 inhibition, to assess the occurrence of possible off-target effects, likely responsible of unwanted toxicities (**Table 3**). We believe that, although cycloguanil display a slightly better profile as far as efficacy, potency and specificity of the antitumor effects, no one of the drugs tested meets, at best, all these features. However, the establishment of a biguanide antitumor class-effect and the identification of possible structural determinants of the antiproliferative activity of this family of drugs, will provide the basis for the design and development of novel compounds endowed with a better pharmacological profile (high potency and low toxicity) which, in combination with already approved cytotoxic drugs [i.e., temozolomide (Valtorta et al., 2017)], might open a new perspective for this still incurable tumor.

**Table 3:** Graphical representation of the pharmacological features of the different biguanides tested as antitumor agents, evaluating EFFICACY (maximal antiproliferative effects), POTENCY (IC_50_), and SELECTIVITY (toxic effects induced via a mechanism independent from CLIC1 inhibition, occurring in cells different from GSCs).

	COMPOUND					
	*Metformin*	*Phenformin*	*Cycloguanil*	*Moroxydine*	*Proguanil*
**EFFICACY**	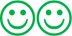	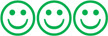	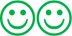	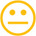	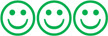
**POTENCY**	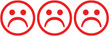	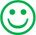	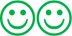	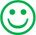	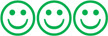
**SELECTIVITY**	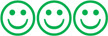	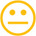	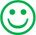	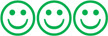	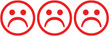

*Green symbols mean high effects (bigger depending on the number of symbols), yellow symbols represent moderate effects, and red symbols indicate low activity.*

## Author Contributions

TF and MM conceived and designed the experiments. FB, RW, AP, IV, CM, MC, AS, AB, AD, and LV performed the experiments. AD and MT contributed reagents, materials, and analysis tools. TF, FB, AD, LV, and MM wrote the manuscript. All authors analyzed the data.

## Conflict of Interest Statement

The authors declare that the research was conducted in the absence of any commercial or financial relationships that could be construed as a potential conflict of interest.
