# Supplementary material for: Inhibition of Chloride Intracellular Channel 1 (CLIC1) as Biguanide Class-Effect to Impair Human Glioblastoma Stem Cell Viability
Source: Front Pharmacol. 2018 Aug 21;9:899. doi: 10.3389/fphar.2018.00899 (PMC6110922; doi:10.3389/fphar.2018.00899)

Supplementary Material

**INHIBITION OF CHLORIDE INTRACELLULAR CHANNEL 1 (CLIC1) AS BIGUANIDE CLASS-EFFECT TO IMPAIR HUMAN GLIOBLASTOMA STEM CELL VIABILITY**

**Federica Barbieri^1^, Roberto Würth^1^^, Alessandra Pattarozzi^1^, Ivan Verduci^2^, Chiara Mazzola^2^, Michele Tonelli^3^, Maria Grazia Cattaneo^4^, Agnese Solari^1^, Adriana Bajetto^1^, Antonio Daga^5^, Lucia M. Vicentini^4^, Michele Mazzanti^2^* and Tullio Florio^1,5^***

*^1^ Sezione di Farmacologia, Dipartimento di Medicina Interna & Centro di Eccellenza per la Ricerca Biomedica (CEBR), Università di Genova, 16132, Genova, Italy*

*^2^ Dipartimento di Bioscienze, Università degli Studi di Milano, 20133 Milano, Italy*

*^3^ Dipartimento di Farmacia, Università di Genova, 16132 Genova, Italy*

*^4^ Dipartimento di Biotecnologie Mediche e Medicina Traslazionale, Università degli Studi di Milano, 20129 Milano, Italy*

*^5^ IRCCS Ospedale Policlinico San Martino, 16132, Genova, Italy*

*** Correspondence: tullio.florio@unige**.**it** and **michele.mazzanti@unimi.it**

**^** *Present address: Division of Stem Cells and Cancer, Deutsches Krebsforschungszentrum (DKFZ), 69120 Heidelberg, Germany*

# Supplementary Figures and Tables

## Supplementary Figures

**SUPPLEMENTARY FIGURE 1**

Dose-response curves of the antiproliferative effect of biguanide derivatives on GBM CSC viability, evaluated by MTT assay after 48 h of treatment. Curves depict the effect of the different drugs on CSC cultures derived from individual human GBM. Data represent the mean ± S.E.M of at least n=3 independent experiments, performed in quadruplicate on each culture. Metformin effects were tested in GBM1, 2, 3, 4, 5, 6, and 7, phenformin and cycloguanil in GBM1, 2, 3, 4, 5, and 6, moroxydine in GBM1, 2, 3, 4, and 6, while proguanil in GBM2, 3, and 4.

**A**

**B**

**C**

**D**

**E**

**SUPPLEMENTARY FIGURE 2**

Sox2

β-actin

35kDa

GBM2

GBM1

CTR

MET

PHEN

MOR

CG


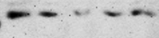

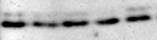

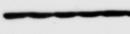

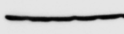


35kDa

CTR

MET

PHEN

MOR

CG

**A**

**B**

50kDa

50kDa

**Biguanides treatment reduce Sox2 protein levels in GSCs.**

A) Representative immunoblots of Sox2 expression in GBM1 and GBM2 GSCs treated with metformin (MET 10mM), phenformin (PHEN, 250 µM), cycloguanil (CG, 200 µM), and moroxydine (MOR, 1 mM) for 24h. β-actin was used as protein loading control.

B) Densitometric analysis of Sox2 expression in which the data were normalized against β-actin levels and reported as percentage of control values (CTR).

**SUPPLEMENTARY FIGURE 3**


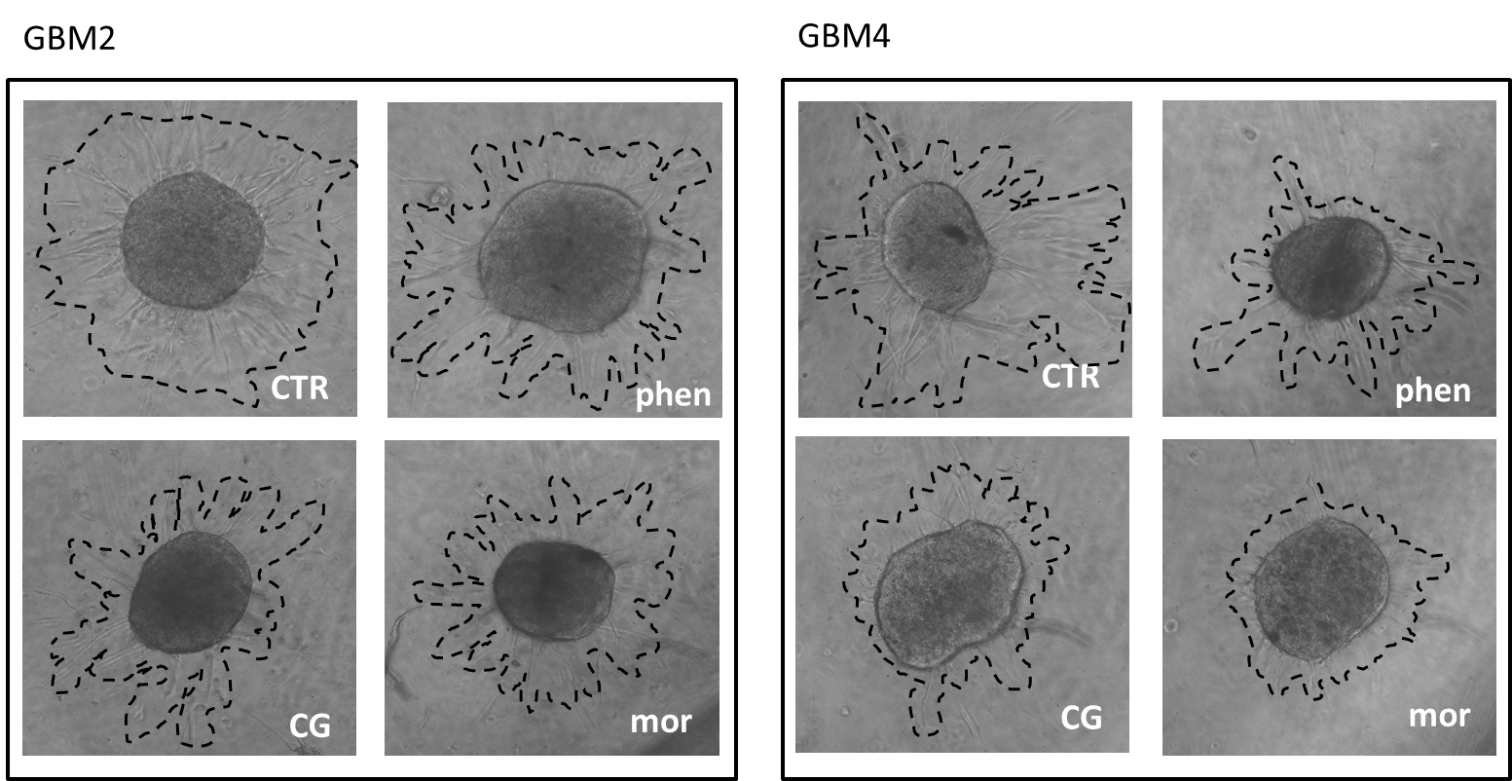


Representative images of GSC sprouting out from spheroids of control or biguanide-treated GBM2 and GBM4 cultures. Control cells: CTR; phenformin: phen (500 µM); cycloguanil: CG (100 µM); moroxydine: mor (1 mM).

**SUPPLEMENTARY FIGURE 4**

Membrane current from GBM4 has been elicited by a 100 mV voltage step, from -40 to +60 mV, 800 msecs in duration. Stimulus has been delivered every 10 secs to generate a current amplitude time course. CLIC1 current inhibition is calculated as ratio of the effect induced by each compound and the inhibition observed after IAA94 (100 μM) treatment that represents the residual CLIC1 activity. For each compound are shown experimental data at two different concentrations reported on the top of each current time-course (left panels). The right panels depict box chart plots showing the average inhibition of CLIC1 current, calculated as ratio between the compound and IAA94 sensitive currents. Number of cells used for the statistics range from 4 to 8 for each compound concentration (empty circles next to box charts). Unpaired t-test: *p<0.05, **p<0.01, ***p<0.001 *vs.* CTR cells.

**SUPPLEMENTARY FIGURE 5**

**CLIC1 down-regulation decreases the sensitivity of glioblastoma stem cells to biguanides.**

**
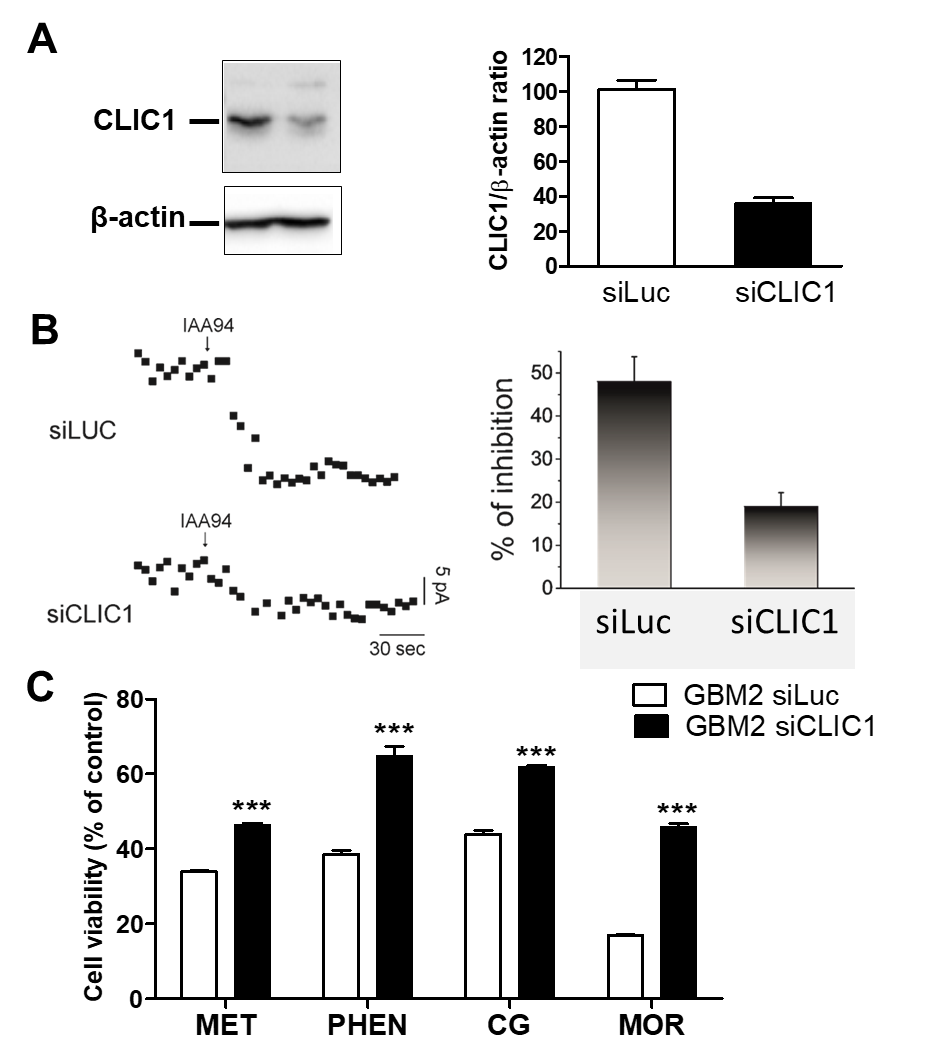
**

GBM2 CSCs in which CLIC1 was down-regulated (siCLIC1) or transfected with control vector (siLuc, as a silencing control) were tested for CLIC1 expression and activity (A, B), and for the antiproliferative efficacy of metformin (MET, 10mM), phenformin (PHEN, 250µM), cycloguanil (CG, 100 µM), moroxydine (MOR, 1mM) (C).

1. Western blot showing that in siCLIC1 cells, CLIC1 protein content is reduced (left panel) and quantified by the densitometric analysis as CLIC1/β-actin ratio.
2. Time course of GBM2 membrane current. In the left panels is reported that IAA94 (100 μM) is able to inhibit the CSC current interfered with control plasmid (top) and for CLIC1 protein (bottom). In the right panel is reported the quantification of current block. Inhibition is expressed as a percentage of absolute membrane current values: siLUC 48±5.8 (n=5), siCLIC1 19±3.2 (n=5).
3. Down-regulation of CLIC1 expression significantly reduces the effects biguanides on CSC viability after 48h of treatment, evaluated by MTT assay. Bars represent the percentage of cell viability in siLuc (white bars) and siCLIC1 (black bars) cells *vs*. respective vehicle-treated controls set as 100%. (ANOVA p=0.001; Tukey’s post-test, ***p<0.001).

**SUPPLEMENTARY FIGURE 6**

**GBM1**

**35KDa**

**25KDa**


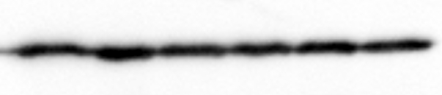


**GBM2**

**35KDa**

**25KDa**


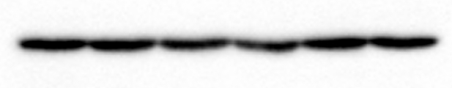


**GBM3**


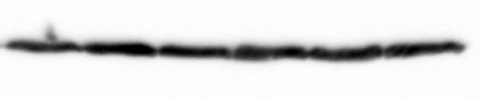


**35KDa**

**25KDa**

**50KDa**

**35KDa**

**50KDa**

**35KDa**

**50KDa**

**35KDa**


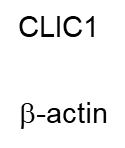


CLIC1

β-actin

CLIC1

β-actin

CTR

MET

PHEN

CG

MOR

IAA94

Effect of biguanide treatment on CLIC1 expression in GSCs from GBM1, 2 and 3. Treatment were performed for 24 hours with: vehicle (controls, CTR), metformin (MET, 10mM), phenformin (PHEN, 250 μM), cycloguanil (CG, 200 μM), moroxydine (MOR, 500 μM), and IAA94 (100 μM). No significant changes in CLIC1 expression were induced by any treatment after normalization for β-actin expression.

**SUPPLEMENTARY FIGURE 7**

**
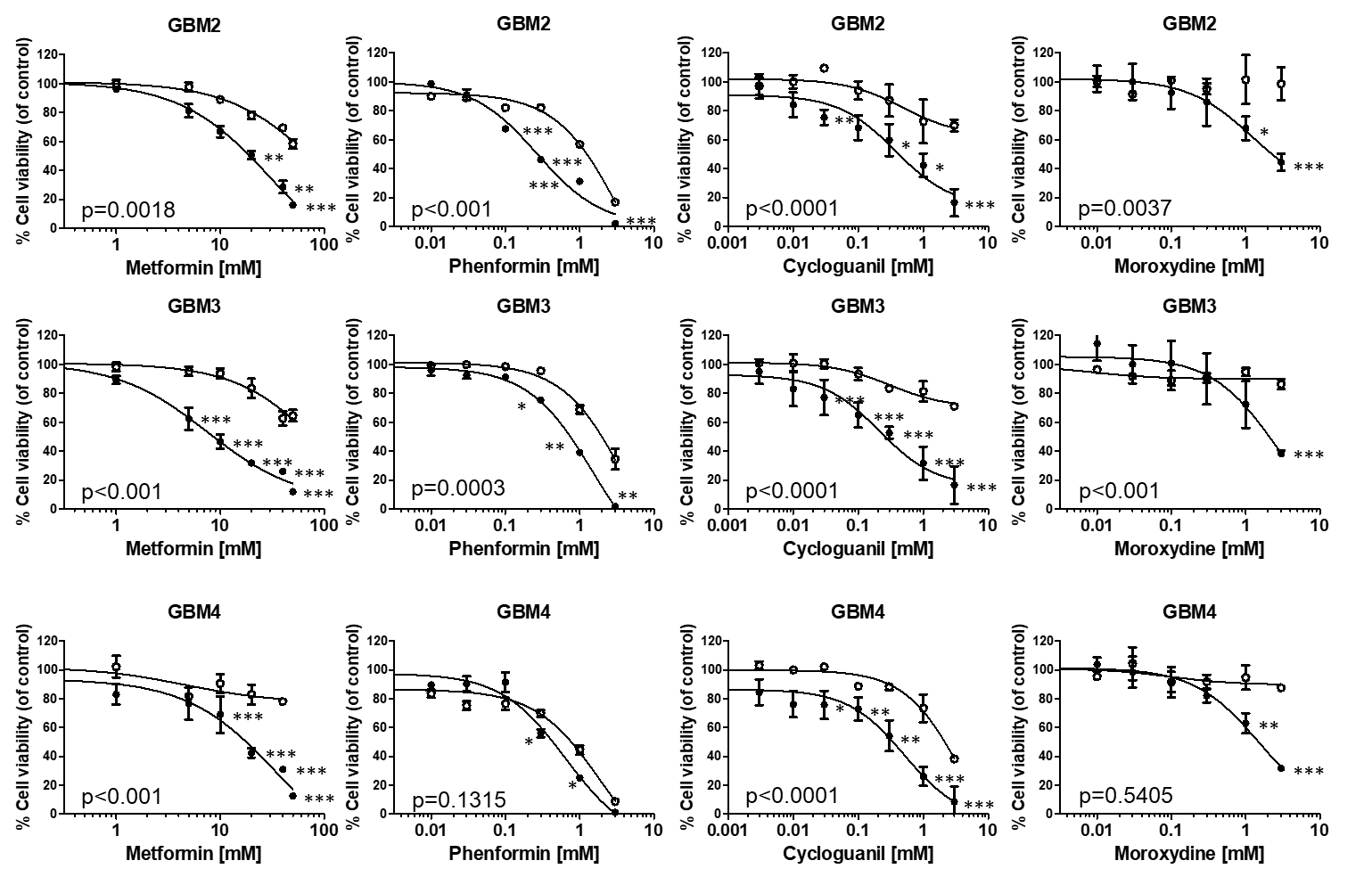
**

Representative dose-response curves depicting the antiproliferative activity of metformin, phenformin, cycloguanil, and moroxydine in GSCs (black circles) and their differentiated counterpart (white circles) derived from GBM2, 3, and 4. Data were compared by two-way ANOVA and the resulting *p* values reported in each graph indicate whether cell type has an effect on response to the drug (i.e. different sensitivity to the drugs between GSCs and differentiated cells). Since the treatment had a different effect in GSCs and differentiated GBM cells, the post-hoc Bonferroni test was used to determine at which concentration the treatment is statistically significant. (* =p<0.05; ** =p<0.01; ***=p<0.001). Data are reported as mean ± SEM (n= 3), and expressed as % on vehicle treated cells.

## Supplementary Tables


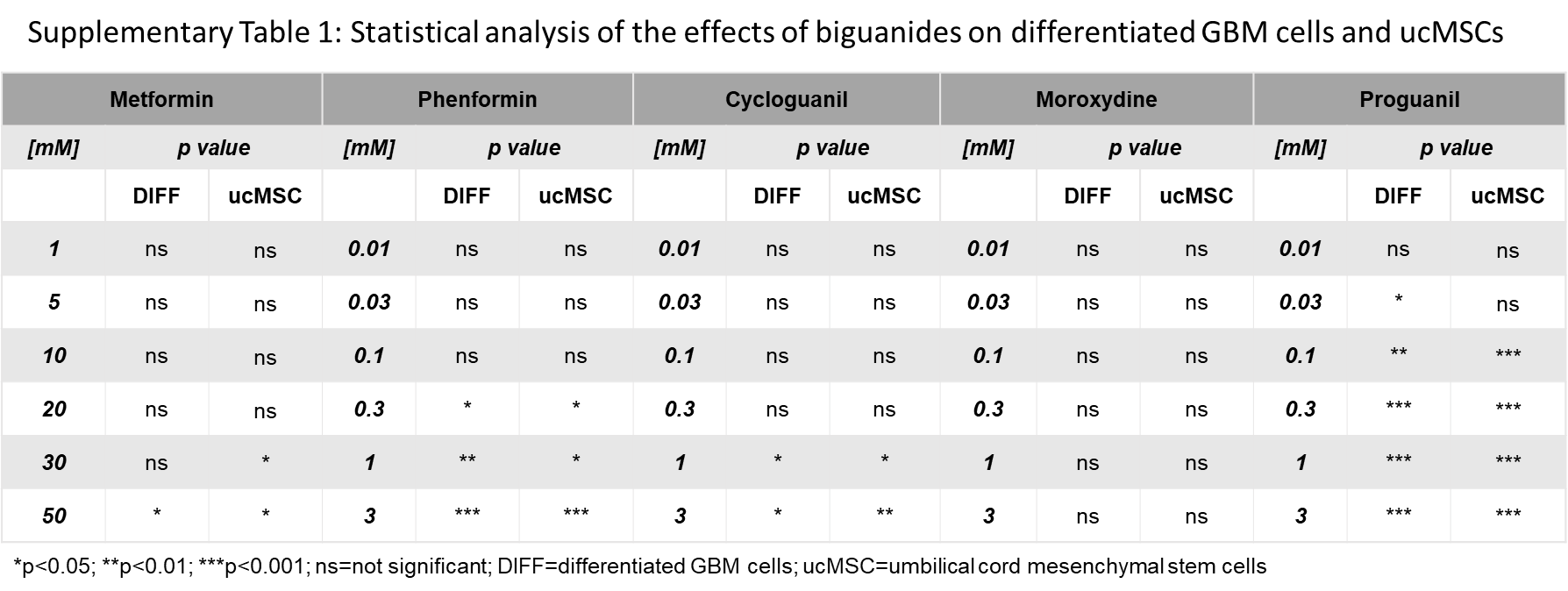

Supplement: Supplementary file 1 [file Table_1.docx]
